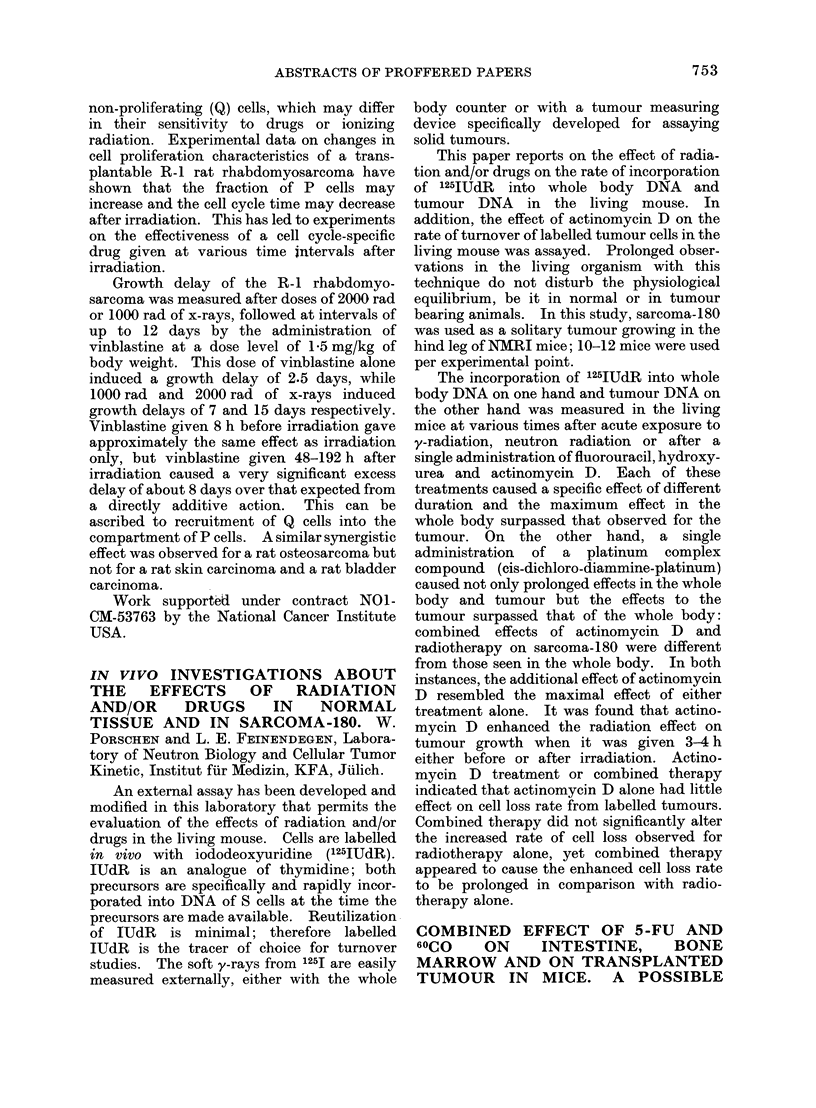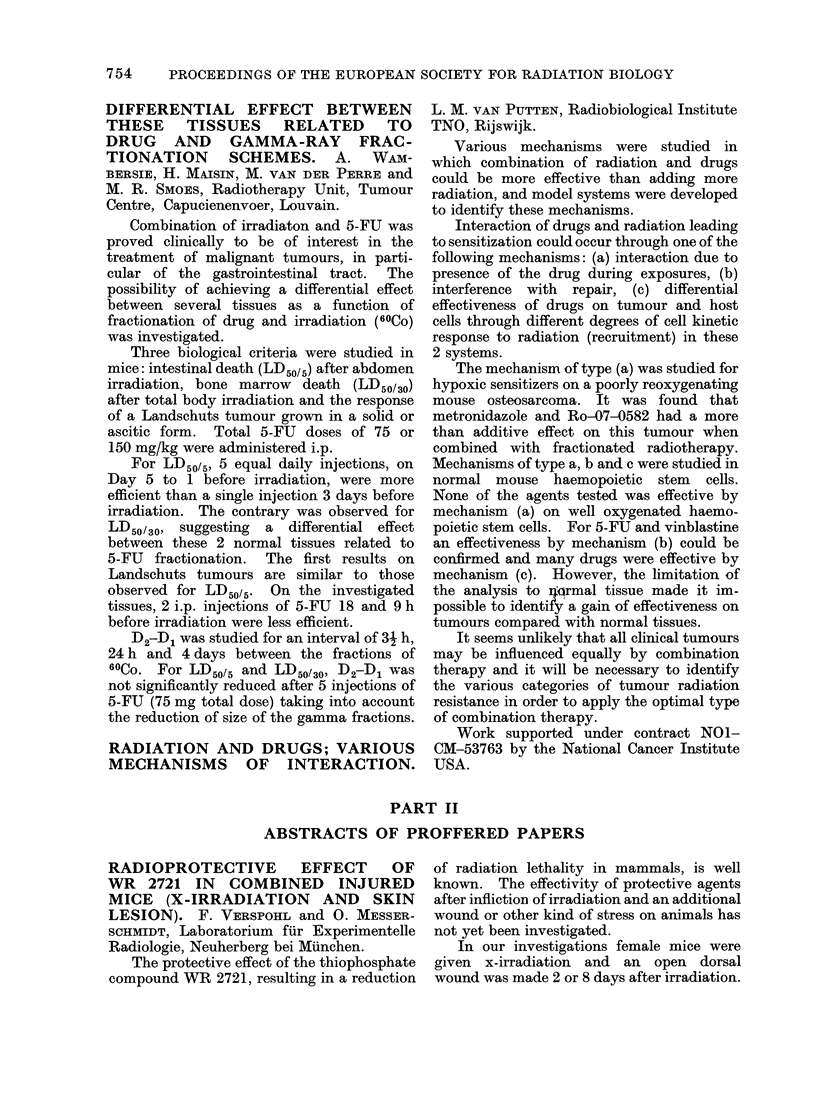# Proceedings: Combined effect of 5-FU and 60Co on intestine, bone marrow and on transplanted tumour in mice. A possible differential effect between these tissues related to drug and gamma-ray fractionation schemes.

**DOI:** 10.1038/bjc.1975.297

**Published:** 1975-12

**Authors:** A. Wambersie, H. Maisin, M. van der Perre, M. R. Smoes


### COMBINED EFFECT OF 5-FU AND $^{60}$CO ON INTESTINE, BONE MARROW AND ON TRANSPLANTED TUMOUR IN MICE. A POSSIBLE

**DIFFERENTIAL EFFECT BETWEEN THESE TISSUES RELATED TO DRUG AND GAMMA-RAY FRACTIONATION SCHEMES.** A. Wambersie, H. Maisin, M. van der Perre and M. R. Smoes, Radiotherapy Unit, Tumour Centre, Capucienenvoer, Louvain.

Combination of irradiaton and 5-FU was proved clinically to be of interest in the treatment of malignant tumours, in particular of the gastrointestinal tract. The possibility of achieving a differential effect between several tissues as a function of fractionation of drug and irradiation ($^{60}Co$) was investigated.

Three biological criteria were studied in mice: intestinal death ($LD_{50/5}$) after abdomen irradiation, bone marrow death ($LD_{50/30}$) after total body irradiation and the response of a Landschuts tumour grown in a solid or ascitic form. Total 5-FU doses of 75 or 150 mg/kg were administered i.p.

For $LD_{50/5}$, 5 equal daily injections, on Day 5 to 1 before irradiation, were more efficient than a single injection 3 days before irradiation. The contrary was observed for $LD_{50/30}$, suggesting a differential effect between these 2 normal tissues related to 5-FU fractionation. The first results on Landschuts tumours are similar to those observed for $LD_{50/5}$. On the investigated tissues, 2 i.p. injections of 5-FU 18 and 9 h before irradiation were less efficient.

$D_2$–$D_1$ was studied for an interval of $3\frac{1}{2}$ h, 24 h and 4 days between the fractions of $^{60}Co$. For $LD_{50/5}$ and $LD_{50/30}$, $D_2$–$D_1$ was not significantly reduced after 5 injections of 5-FU (75 mg total dose) taking into account the reduction of size of the gamma fractions.